# Mitochondrial Dysfunction in Alzheimer’s Disease and Mitochondria-Targeted Therapeutics

**DOI:** 10.3390/cells15110990

**Published:** 2026-05-28

**Authors:** Jasbir Bisht, Priyanka Rawat, Andrew C. Shin, Vijay Hegde

**Affiliations:** 1 Obesity and Metabolic Health Laboratory, Department of Nutritional Sciences, Texas Tech University, Lubbock, TX 79409, USA; jasbir.bisht@ttu.edu (J.B.); prrawat@ttu.edu (P.R.); 2 Neurobiology of Nutrition Laboratory, Department of Nutritional Sciences, Texas Tech University, Lubbock, TX 79409, USA; andrew.shin@ttu.edu

**Keywords:** Alzheimer’s disease, mitochondrial dysfunction, oxidative stress, neurodegenerative disease, mitochondrial therapeutics, neuronal loss

## Abstract

**Highlights:**

**What are the main findings?**

- Mitochondrial dysfunction is an early and important contributor to Alzheimer’s disease (AD), influencing oxidative stress, impaired energy metabolism, synaptic dysfunction, and neuronal loss.
- Mitochondria-targeted therapies, including antioxidants, mitophagy enhancers, and metabolic modulators, show promising neuroprotective and cognitive benefits in preclinical AD models.

**What are the implications of the main findings?**

- Targeting mitochondrial dysfunction may provide a disease-modifying strategy for AD by addressing multiple pathogenic mechanisms simultaneously.
- Future progress will depend on early intervention, improved biomarkers, and precision-based mitochondrial therapies to enhance clinical translation in AD.

**Abstract:**

Alzheimer’s disease (AD) is the most prevalent form of dementia and is characterized by progressive cognitive decline due to the loss of neurons. The accumulation of extracellular senile plaques (Aβ) and intracellular tau neurofibrillary tangles (NFTs) is a key pathological feature of AD. Mitochondrial dysfunction is implicated in all key AD pathologies, whether as a cause or a consequence of disease progression. Growing evidence indicates that mitochondrial impairment plays a central role in AD pathogenesis by disrupting cellular homeostasis, promoting oxidative stress, and contributing to progressive neuronal death. Therefore, targeting mitochondria may offer promising insights into the development of disease-modifying therapies. In this review, we summarize current evidence on the role of mitochondrial dysfunction in the pathophysiology of AD and on its therapeutic potential.

## 1. Introduction

Alzheimer’s disease (AD), an age-related, progressive, neurodegenerative disease, is the most prevalent form of dementia in older adults. According to Alzheimer’s disease figures and facts 2025, currently, an estimated 7.2 million Americans aged 65 have AD. Without medical advances to prevent, slow, or cure AD, this number will surpass 13.8 million by 2060. The impact of AD can be profoundly distressing for individuals who are affected by this disease, as well as their families and caregivers [1]. AD is described as a result of memory loss and a range of cognitive deficits. Accumulation of amyloid beta Aβ and neurofibrillary tangles due to hyperphosphorylated tau is a central pathological feature of the AD brain [2,3]. The aggregation of these pathological markers leads to various consequences, including, but not limited to, mitochondrial impairments, oxidative stress, glial cell activation, neuroinflammation, and dysregulation of microRNAs, which are associated with the gradual deterioration of synaptic transmission and loss of neurons, which in turn influence the progression of cognitive deficits [4,5]. Although the exact cause and underlying pathophysiological mechanisms of AD have yet to be fully determined, the accumulation or improper removal of toxic Aβ in the brain is believed to play a crucial role in advancing the disease progression [6,7].

**Figure 1:**
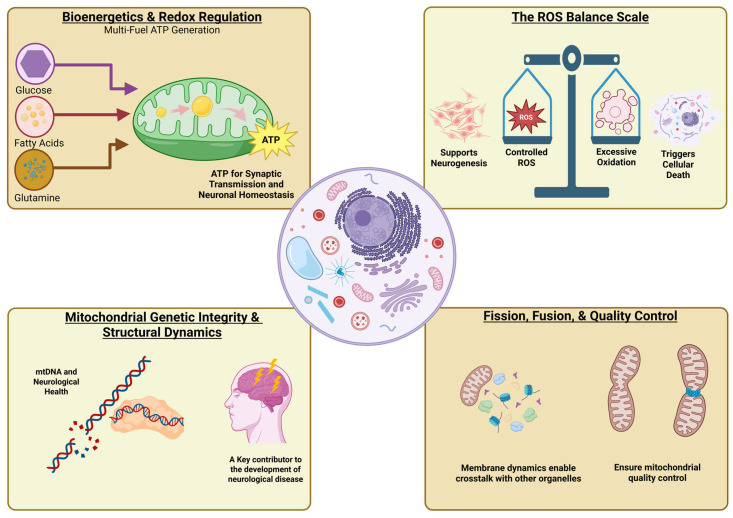
Schematic representation of the key functional domains of mitochondrial homeostasis, including bioenergetics, redox balance, mt DNA integrity, and fission–fusion quality control.

Mitochondria have appeared as the main regulators of neuronal survival and function, playing vital roles in ATP production, reactive oxygen species (ROS), mtDNA (which encodes electron transport chain proteins, and its dysfunction leads to impaired energy production and oxidative stress), membrane dynamics, calcium homeostasis, redox signaling, and apoptotic regulation (Figure 1) [8]. Neurons are particularly susceptible to mitochondrial impairment due to their high metabolic demands, polarized morphology, and dependence on a defined mitochondrial distribution at synapses [9,10]. Several studies suggest that mitochondrial anomalies occur early in AD pathogenesis and may contribute to amyloid and tau pathology, positioning mitochondrial dysfunction as a potential initial phenomenon. Conversely, amyloid-beta and tau deposition exacerbate mitochondrial dysfunction, supporting a bidirectional relationship.

The mitochondrial cascade hypothesis of AD proposes that inherited and age-related mitochondrial alterations lead to impaired bioenergetics, excessive ROS production, altered mitochondrial dynamics, defective mitophagy, and loss of synaptic function, contributing to neuronal vulnerability and progressive cognitive decline [9,10]. These mitochondrial disparities correspond with classical AD pathologies by promoting the accumulation of Aβ, hyperphosphorylation of tau, and neuroinflammatory responses, thereby creating a self-propagating progression of cellular dysfunction [11,12].

Given the important role of mitochondria in integrating metabolic, oxidative, and synaptic pathways, mitochondrial dysfunction represents a promising therapeutic target for AD [10]. Developments in mitochondria-targeted antioxidants, metabolic modulators, mitophagy enhancers, gene-based therapies, and mitochondrial transplantation have opened new opportunities for disease-modifying interventions. This review critically examines the evidence supporting mitochondrial dysfunction as a potential contributor to AD pathogenesis. It provides an in-depth evaluation of emerging mitochondrial-targeted therapeutic approaches, highlighting their mechanistic basis and translational potential.

## 2. Evidence of Mitochondrial Dysfunction in AD

Swerdlow and Khan proposed the mitochondrial cascade hypothesis in 2004 and explained that mitochondrial dysfunction plays a significant role in AD [13,14]. Although this hypothesis has not been conclusively validated in all AD models or clinical settings, an extensive body of evidence suggests prevalent mitochondrial abnormalities in AD brains, supporting a critical role for mitochondrial impairment in AD progression and pathogenesis [15].

**Figure 2:**
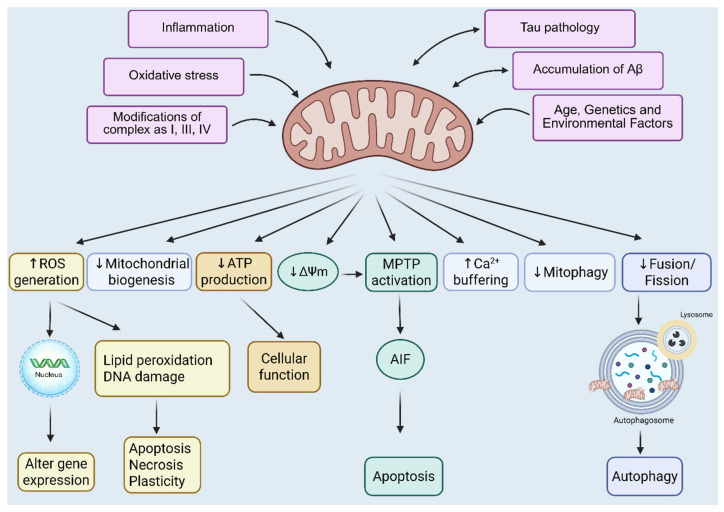
Schematic representation of mitochondrial impairment in AD, including Aβ accumulation, tau pathology, inflammation, oxidative stress, and ETC complex alterations. These changes impair ATP production, mitochondrial membrane potential, calcium buffering, mitophagy, and the balance between fusion and fission, while increasing ROS generation and mPTP activation. Together, these disturbances contribute to oxidative damage, cellular dysfunction, and neuronal death.

Numerous aspects of mitochondrial integrity and function are disrupted in AD, including altered mitochondrial morphology, reduced membrane potential, impaired oxidative phosphorylation, enhanced ROS production, defective calcium buffering, mtDNA impairment, dysfunctional mitochondrial biogenesis, abnormal axonal transport of mitochondria, and defective mitophagy. All these irregularities indicate a complete failure of mitochondrial quality control and bioenergetic homeostasis in AD rather than individual defects within a single pathway (Figure 2) [15,16].

Neurons are extremely susceptible to mitochondrial damage due to their high energy requirement, polarized morphology, and dependence on efficient mitochondrial trafficking to synaptic compartments. Consequently, even minor mitochondrial dysfunction can substantially influence synaptic transmission, plasticity, and neuronal resilience [17]. Mitochondrial abnormalities often occur before amyloid plaque deposition and NFT formation, according to mounting data from AD individuals, transgenic AD mouse models, and cell culture [18,19,20]. This finding suggests that mitochondrial dysfunction may act as an early event rather than a downstream consequence of AD pathology. These findings also support that mitochondrial dysfunction is a persistent and early feature of AD, affecting several interconnected mitochondrial pathways. This extensive impairment provides a mechanistic background that links metabolic failure, oxidative stress, synaptic dysfunction, and neuronal loss, thereby aligning mitochondria as a central player in AD pathogenesis and a rational target for therapeutic intervention.

### 2.1. Altered Energy Metabolism in AD

Normally, glucose is the primary energy source for brain cells. In a resting awake state, the brain uses 20% of the body’s oxygen, more than 25% of the body’s glucose, and weighs an average of 2% of the body’s total weight [10]. One of the earliest and most consistent metabolic abnormalities observed in AD is a pronounced cerebral hypometabolic state, characterized by impaired glucose uptake and consumption. Neurons depend extremely on glucose-driven mitochondrial oxidative phosphorylation to fulfill their intense high-energy demands [21,22]. In AD, altered glucose metabolism and impaired mitochondrial bioenergetics create a chronic energy shortage that compromises synaptic function and contributes to neurodegeneration. Mitochondrial dysfunction directly alters energy metabolism by reducing the efficiency of the electron transport chain (ETC), diminishing tricarboxylic acid (TCA) cycle activity, and affecting cellular bioenergetic pathways. Premature electron leakage at complexes I and III results in increased production of ROS, further damages mitochondrial components, and exacerbates bioenergetic failure [23,24]. This imbalance between energy production and oxidative stress leads to diminished cytochrome c oxidase activity and progressive decline of mitochondrial respiratory competence in AD brains [24].

Neuroimaging studies using positron emission tomography (PET) have consistently confirmed a 20–30% decline in cerebral glucose metabolism in individuals with AD compared to cognitively healthy individual controls [25]. Hypometabolism is evidently examined in brain regions critical for learning and memory, including the hippocampus, posterior cingulate cortex, and temporal and parietal lobes. Notably, these metabolic deficits are seen early, often preceding clinical symptoms and structural brain atrophy, and progressively spread to the frontal and occipital regions as cognitive impairment develops [26,27]. At the molecular level, impaired glucose transport and insulin signaling further contribute to cerebral energy shortage in AD [28]. Glucose uptake into the brain is mainly facilitated by glucose transporter 1 (GLUT1) at the blood–brain barrier and astrocytes, while neuronal glucose uptake depends mainly on glucose transporter 3 (GLUT3) [29]. Aging and AD are associated with lowered expression of neuronal glucose transporters, particularly GLUT3 and glucose transporter 4 (GLUT4), and with reduced glucose availability to neurons even with preserved vascular delivery [30]. These deficits are exacerbated by brain insulin resistance, characterized by reduced insulin receptor expression and impaired downstream signaling pathways, further disrupting neuronal glucose utilization and mitochondrial function. Insulin modulates neuronal and glial cell activity, thereby altering mood, cognition, and behavior. Additionally, due to its role in promoting neuronal health, insulin may protect against AD. Brain insulin resistance also overlaps with amyloid pathology by impairing Aβ clearance and promoting its accumulation. Insulin competitively inhibits insulin-degrading enzyme (IDE), reducing Aβ breakdown, while altered insulin signaling interferes with low-density lipoprotein receptor-related protein 1 (LRP1) trafficking, damaging Aβ clearance [31]. These processes strengthen a vicious cycle in which metabolic dysfunction, mitochondrial impairment, and amyloid pathology reciprocally exacerbate one another. These findings support the concept of AD as a state of chronic cerebral energy failure mediated by the combination of impaired glucose metabolism, insulin resistance, and mitochondrial dysfunction. This bioenergetic collapse leads to evident neurodegeneration.

### 2.2. Impaired Mitochondrial Dynamics in AD

Mitochondrial dynamics, governed by coordinated fusion and fission processes, are essential for maintaining mitochondrial integrity, distribution, and function in neurons. Through continuous remodeling, these processes allow mitochondria to adapt to metabolic demands, facilitate mitochondrial transport along axons and dendrites, and support synaptic activity. Given the polarized morphology and high energy requirements of neurons, precise regulation of mitochondrial dynamics is particularly critical for neuronal survival and plasticity [17]. Mitochondrial fusion promotes the combination of mitochondrial contents, including proteins, lipids, and mtDNA, thereby protecting the function of mitochondria and compensating for localized damage [32]. This process is facilitated by the dynamin-related GTPases mitofusin 1 (MFN1) and mitofusin 2 (MFN2), which regulate outer mitochondrial membrane fusion, and optic atrophy 1 (OPA1), which controls inner membrane fusion and cristae organization [33,34]. In contrast, mitochondrial fission facilitates mitochondrial replication, distribution, and quality control by isolating damaged mitochondria for removal via mitophagy. Fission is mainly facilitated by dynamin-related protein 1 (DRP1), which is recruited to the mitochondrial outer membrane where it constricts and divides mitochondria in a GTP-dependent manner [35].

In AD, this balance between fusion and fission is disrupted, resulting in extreme mitochondrial fission. Numerous studies in postmortem human AD brains, transgenic mouse models, and neuronal culture have demonstrated increased DRP1 activity, accompanied by reduced expression of fusion proteins such as MFN1, MFN2, and OPA1 [36,37]. This shift toward increased fission and diminished fusion represents an early and consistent feature of AD pathology, preceding significant neuronal loss and showing a strong relationship with synaptic dysfunction [37,38,39]. Excessive mitochondrial fission has profound functional consequences. Fragmented mitochondria demonstrate impaired bioenergetic capacity, reduced calcium buffering, and reduced efficiency in transport to synaptic terminals [10]. As synapses are highly energy-dependent structures, compromised mitochondrial trafficking and localization result in defective synaptic transmission, reduced plasticity, and increased vulnerability to excitotoxic and oxidative stress [17]. Moreover, fragmented mitochondria are more susceptible to oxidative damage and are less capable of maintaining normal cristae architecture, further exacerbating mitochondrial impairment in AD [40,41]. Abnormal mitochondrial dynamics are also combined with other pathogenic pathways in AD. Aβ and hyperphosphorylated tau have been shown to directly interact with mitochondrial fission and fusion proteins, particularly DRP1, promoting excessive fission and boosting mitochondrial fragmentation [42,43]. This interaction creates a feed-forward loop in which AD pathology accelerates mitochondrial dysfunction, which in turn amplifies synaptic failure and neuronal loss. These findings suggest impaired mitochondrial dynamics as a critical contributor to AD pathogenesis by disrupting mitochondrial morphology, transport, and function.

### 2.3. Impaired Mitochondrial Biogenesis in AD

Mitochondrial biogenesis is a strictly regulated process that maintains an adequate pool of functional mitochondria, particularly in energy-demanding cells such as neurons [44]. By regulating the synthesis of mitochondrial proteins, the replication of mtDNA, and the assembly of respiratory chain complexes, mitochondrial biogenesis promises sustained bioenergetic function and redox balance. In neurons, continuous mitochondrial renewal is required to compensate for mitochondrial damage and support synaptic activity. The transcriptional regulation of mitochondrial biogenesis is primarily mediated by peroxisome proliferator-activated receptor-γ coactivator-1α (PGC-1α), a master regulator that integrates metabolic signals, oxidative stress responses, and mitochondrial function [45,46,47]. PGC-1α coactivates nuclear respiratory factors (NRF1 and NRF2), which, in turn, regulate the expression of mitochondrial transcription factor A (TFAM), a significant driver of mtDNA transcription and replication [48,49,50]. Through this signaling network, PGC-1α coordinates mitochondrial biogenesis with cellular energy demands and antioxidant defense processes [51]. Compelling evidence shows that mitochondrial biogenesis is markedly impaired in AD. Reduced PGC-1α expression has been consistently observed in postmortem AD brains, and experimental studies in transgenic mouse models support early suppression of PGC-1α signaling even before substantial Aβ deposition [52,53]. Reductions in PGC-1α, NRF1, NRF2, TFAM, and upstream regulatory factors such as cAMP response element-binding protein (CREB) and protein kinase-A have been detected in young 3xTg-AD mice, suggesting that defects in mitochondrial biogenesis arise early in disease progression and lead to neuropathological changes [54]. Impaired mitochondrial biogenesis in AD occurs in parallel with defective Mitophagy, resulting in a net loss of functional mitochondria.

While damaged mitochondria accumulate due to inefficient clearance, the reduced capacity to produce new mitochondria further exacerbates bioenergetic failure. This imbalance between mitochondrial removal and replacement leads to reduced ATP production, increased oxidative stress, and enhanced neuronal vulnerability. Due to the limited regenerative capacity of neurons, prolonged inhibition of mitochondrial biogenesis has particularly detrimental consequences for synaptic maintenance and neuronal survival [55]. Significantly, PGC-1α signaling also crosses directly with classical AD pathologies. PGC-1α has been shown to reciprocally regulate Beta-site APP cleaving enzyme 1 (BACE1) in vitro and in vivo, acting together with Sirtuin 1 (SIRT1)-mediated deacetylation of Peroxisome proliferator-activated receptor gamma (PPARγ). These coordinated mechanisms are essential for controlling Aβ production in AD [56].

Additionally, PGC-1α plays a critical role in regulating mitochondrial antioxidant defenses, and its downregulation leads to increased ROS accumulation and oxidative damage in AD neurons [57,58]. Furthermore, these findings support impaired mitochondrial biogenesis as a significant early event in AD pathogenesis and, by limiting mitochondrial renewal under increasing metabolic stress, suppress PGC-1α-mediated biogenesis, amplifying mitochondrial dysfunction, and contributing to AD pathology.

### 2.4. Increased Oxidative Stress and Mitochondrial Defects in AD

Oxidative stress is a prominent and early pathological feature of AD and is closely linked with mitochondrial dysfunction [59,60]. It begins with an imbalance between ROS production and the function of cellular antioxidant defenses to neutralize them. The brain is exceptionally susceptible to oxidative damage due to its high oxygen consumption, abundant lipid contents, and comparatively limited antioxidant capacity, making neurons especially vulnerable to redox imbalance. Mitochondria are the primary source of intracellular ROS, with the majority generated as byproducts of oxidative phosphorylation due to electron leakage at complexes I and III of the ETC [61]. In AD, mitochondrial respiratory ineffectiveness exacerbates this process, leading to excessive ROS production. Notably, mitochondria are not only the primary generators of ROS but also their principal targets. ROS-mediated damage to mitochondrial DNA, proteins, and membrane lipids further impairs mitochondrial function, thereby perpetuating a self-reinforcing cycle of oxidative stress and bioenergetic failure [62,63,64].

Postmortem AD brains show elevated levels of oxidized lipids, proteins, and nucleic acids compared with age-matched healthy controls, indicating extensive oxidative damage [65,66]. Among mitochondrial defects, deficiency of cytochrome c oxidase (complex IV) is one of the most consistently reported abnormalities in AD [67]. Reduced complex IV activity impairs ATP production, increases electron leakage, and further increases ROS generation and oxidative injury. These defects contribute directly to neuronal energy failure and synaptic dysfunction. Oxidative stress also plays a critical role in linking mitochondrial dysfunction to tau pathology in AD. Increased ROS levels inhibit protein phosphatase 2A (PP2A), a major tau phosphatase, thereby increasing the activation of glycogen synthase kinase-3β (GSK3β), a key kinase responsible for hyperphosphorylation of tau protein [68]. This shift in kinase–phosphatase balance promotes the formation of NFTs, thereby connecting mitochondrial oxidative stress to one of the central pathological hallmarks of AD [69]. Beyond tau pathology, oxidative damage disturbs synaptic integrity and neuronal signaling by modifying synaptic proteins, impairing calcium homeostasis, and activating apoptotic pathways. During aging, progressive mitochondrial dysfunction further impairs ROS production, while accumulated oxidative damage impairs mitochondrial turnover and quality control mechanisms, including mitophagy [70,71,72,73]. As a result, neurons accumulate dysfunctional mitochondria with diminished respiratory capacity, reinforcing oxidative stress and accelerating neurodegeneration. By acting both upstream and downstream of mitochondrial impairment, oxidative stress amplifies metabolic failure, promotes tau pathology, and reinforces the pathological cascade that drives the neurodegeneration in AD [64].

### 2.5. Impaired Mitophagy in AD

**Figure 3:**
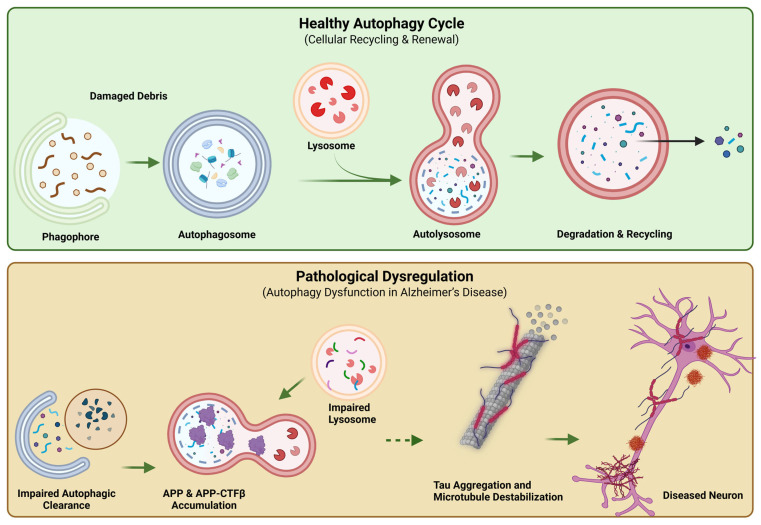
Schematic representation of healthy autophagy and impaired mitophagy in AD, illustrating the role of APP C-terminal beta (APP-CTFβ) accumulation, lysosomal dysfunction, and impaired mitophagic clearance.

Mitophagy is a specialized form of autophagy that selectively removes damaged or dysfunctional mitochondria, thereby preserving mitochondrial quality and cellular homeostasis. In neurons, efficient Mitophagy is essential due to high metabolic demand, limited regenerative capacity, and reliance on long-lived mitochondria [74]. Disruption of this quality control mechanism has emerged as a critical contributor to mitochondrial impairment in AD. The best-characterized mitophagy pathway involves PTEN-induced kinase 1 (PINK1) and the E3 ubiquitin ligase Parkin [75]. Under physiological conditions, PINK1 is rapidly degraded in healthy mitochondria. However, mitochondrial damage and loss of membrane potential stabilize PINK1 on the outer mitochondrial membrane, causing Parkin recruitment and the ubiquitination of mitochondrial proteins [75]. This process signs damaged mitochondria for engulfment by autophagosomes and subsequent degradation through lysosomal fusion (Figure 3) [76].

In AD, multiple lines of evidence indicate that Mitophagy is initiated but fails to proceed efficiently to completion. Electron microscopy studies of postmortem AD brains and transgenic mouse models reveal an accumulation of structurally abnormal mitochondria, characterized by swelling and disrupted cristae [77,78]. Simultaneously, high levels of PINK1, Parkin, and ubiquitinated mitochondrial proteins have been identified in neurons, suggesting an activated yet suspended mitophagy response rather than a lack of mitophagy signaling [79,80,81]. Defective Mitophagy in AD is strongly linked to impaired autophagosome–lysosome fusion and lysosomal dysfunction. Mutations in presenilin 1 (PS1), a key component of the γ-secretase complex, interrupt lysosomal acidification and autophagic flux, leading to the buildup of autophagosomes and unfinished mitochondrial degradation [82,83]. As a result, damaged mitochondria stay within neurons, continuously generating reactive oxygen species and worsening oxidative stress and bioenergetic failure [64]. Impaired mitophagy with other mitochondrial abnormalities observed in AD [79,80,81]. Excessive mitochondrial fission yields fragmented mitochondria that are preferentially targeted for Mitophagy; however, failure of mitochondrial clearance leads to their pathological accumulation. This accumulation further impairs neuronal calcium buffering and metabolic homeostasis, exacerbating overall cellular dysfunction. Furthermore, persistent mitochondrial damage reinforces neuroinflammatory signaling and promotes neuronal vulnerability. Rather than showing insufficient detection of damaged mitochondria, mitophagy impairment in AD arises from disrupted downstream clearance processes. Among the molecular drivers of mitophagy impairment in AD, the Aβ precursor protein C- terminal fragment (APP-CTFβ, also known as C99) has emerged as a key contributor. APP-CTFβ is generated by β-secretase cleavage of APP and serves as the direct precursor to Aβ following further γ secretase processing. APP-CTFβ itself accumulates in AD brains and localizes to mitochondria-associated membranes, where it impairs mitochondrial function, disrupts autophagosomes-lysosome fusion, and inhibits mitophagic clearance of damaged mitochondria, thereby exacerbating mitochondrial dysfunction independently of Aβ pathology [84,85].

### 2.6. Shortage of Neuronal ATP in AD

A sustained decline in mitochondrial ATP production is a critical downstream consequence of mitochondrial dysfunction in AD and a key driver of neuronal and synaptic failure. Neurons depend on mitochondrial oxidative phosphorylation to meet their continuous, high-energy demands, yet they lack substantial energy reserves, such as glycogen or lipid stores [86]. As a result, even modest impairments in mitochondrial ATP generation can have profound consequences for neuronal function and survival. Multiple lines of evidence indicate that ATP production is markedly reduced in the AD brain [64]. Defects in the ETC, reduced activity of cytochrome c oxidase and other respiratory complexes, compromise proton gradient formation and limit ATP synthesis [87]. Excessive ROS production further damages mitochondrial components, exacerbating bioenergetic inefficiency. All these alterations result in a persistent energy deficit that leads to neuronal loss and correlates strongly with cognitive decline. Structural and functional abnormalities of mitochondrial ATP synthase further contribute to ATP shortage in AD. Studies have shown oxidative and nitrative modifications of ATP synthase subunits, as well as altered regulation of the F_1_F_0_-ATP synthase complex, leading to reduced catalytic efficiency [88,89,90]. Oxidative damage to nuclear and mitochondrial DNA encoding ATP synthase components also reduces protein expression, further impairing ATP production [91,92]. These defects focus on ATP synthase dysfunction as a major contributor to neuronal energy failure in AD.

ATP reduction directly affects neuronal signaling and synaptic integrity because synaptic transmission, vesicle recycling, ion pump activity, and maintenance of membrane potential are all highly energy-dependent processes [93]. Reduced ATP availability disrupts calcium homeostasis, impairs axonal transport of organelles and synaptic vesicles, and reduces synaptic plasticity. As a result, synapses become functionally compromised long before neurons undergo irreversible degeneration.

### 2.7. Mitochondrial Calcium Dysregulation in AD

**Figure 4:**
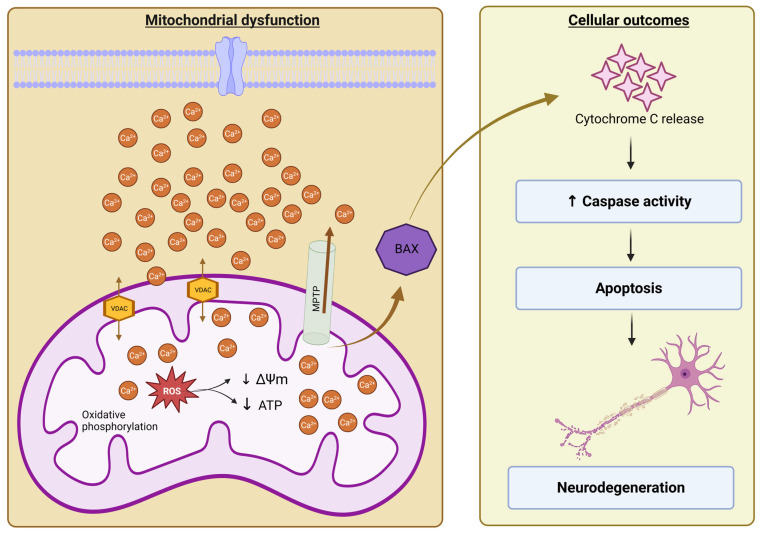
Schematic representation of mitochondrial calcium overload leading to ROS generation, reduced membrane potential, and ATP depletion, disrupting mitochondrial homeostasis. VDAC, voltage-dependent anion channel; MPTP, mitochondrial permeability transition pore; BAX, Bcl-2-associated X protein; ROS, reactive oxygen species ΔΨm, mitochondrial membrane potential.

In mitochondria, calcium levels are strictly regulated. Calcium entry into mitochondria is primarily mediated by the voltage-dependent anion channel (VDAC) on the outer mitochondrial membrane [94], followed by transport across the inner membrane via the mitochondrial calcium uniporter (MCU) [95]. VDAC1, the predominant isoform and the most abundant protein of the outer mitochondrial membrane, regulates metabolite, ATP/ADP, and ion exchange between the cytosol and mitochondria and has been proposed as a key component or regulator of the mitochondrial permeability transition pore (MPTP) [96]. In AD, VDAC1 levels are markedly elevated in postmortem tissues and AD transgenic mouse models, where VDAC1 interacts directly with Aβ and p-tau, promoting mitochondrial calcium overload, MPTP opening, cytochrome c release, and apoptotic signaling [97,98]. When calcium levels within mitochondria increase excessively, this leads to increased ROS production and triggers apoptosis, as observed in AD. Mitochondria help maintain calcium homeostasis by sequestering excess calcium during synaptic activity and releasing it when needed to support key functions, such as ATP production and signal transduction, in healthy neurons [99]. Imbalanced cellular calcium homeostasis is an early and widespread hallmark of the AD brain, and its connection to AD was established decades ago [100,101]. Early imbalance in calcium homeostasis is linked to alterations in calcium-dependent proteases, highlighting their involvement in the preclinical stages of the disease [102]. Additionally, increased basal cytosolic calcium levels and abnormal spontaneous calcium activity have been reported in neurons and astrocytes in AD mouse models [103,104]. The widespread view proposes that extensive calcium overload in neurons leads to neuronal death through several mechanisms, including excessive activation of calcium-dependent kinases and phosphatases, glutamate-induced excitotoxicity, stimulation of calcium-dependent proteases, and mitochondrial calcium deposits that trigger mPTP opening, release of cytochrome c, and activation of caspases and apoptosis [105,106,107]. (Figure 4) Calcium interferes with APP processing, promoting increased Aβ production and NFT formation [108,109]. In vitro studies have shown that mitochondrial calcium overload triggers the neurotoxicity induced by Aβ oligomers, and inhibition of mitochondrial calcium overload provides a novel mechanism of neuroprotection [110]. Another study showed that calcium release from the endoplasmic reticulum depletes cellular GSH and increases ROS, leading to mitochondrial membrane depolarization, which suggests that early Aβ-induced disruptions in ER calcium homeostasis impair mitochondrial function and trigger apoptosis, contributing to neuronal death in AD [111]. In the mouse model of AD, elevated mitochondrial calcium levels contribute to neuronal death [105]. So, targeting reducing the mitochondrial calcium level may offer one of the treatment strategies for AD.

## 3. Therapeutic Interventions for Mitochondrial Dysfunction

Several therapeutic strategies targeting mitochondrial dysfunction have emerged as promising approaches for AD, ranging from mitochondria-targeted antioxidants and small molecules to gene therapy, mitochondrial transplantation, and mitophagy enhancers. The main compounds and their reported effects in AD are summarized in Table 1.

### 3.1. Mitochondria-Targeted Antioxidant Therapies

Given that mitochondria are both a prevalent source and a significant target of ROS, strategies that deliver antioxidants directly to mitochondria have gained substantial attention as potential disease-modifying interventions in AD. Unlike conventional antioxidants, which often show limited benefit due to poor mitochondrial penetration, mitochondria-targeted compounds are designed to accumulate within mitochondria, thereby improving local ROS buffering, preserving mitochondrial membrane integrity, and stabilizing bioenergetic function [112].

#### 3.1.1. MitoQ

MitoQ is a mitochondria-targeted ubiquinone derivative that accumulates in mitochondria and has shown neuroprotective effects in AD animal models. In transgenic 3xTg-AD mice, chronic MitoQ administration improved cognitive behavior, reduced oxidative stress, inflammation, caspase activation, and Aβ42 levels, with behavioral outcomes approaching those of wild-type controls in spatial memory testing [113]. Additionally, in another study, MitoQ-treated 3xTg-AD mice preserved memory compared with untreated mice and reduced oxidative stress, synapse loss, astrogliosis, microglial cell proliferation, Aβ accumulation, caspase activation, and hyperphosphorylation of tau [114]. MitoQ has also been evaluated in clinical settings outside AD, including Parkinson’s disease, supporting its general translational feasibility, although robust clinical efficacy for neurodegeneration remains to be established [115,116,117].

#### 3.1.2. SkQ1

Mitochondria-targeted plastoquinone derivatives such as SkQ1 have also demonstrated neuroprotective effects. In the AD rat model, SkQ1 treatment during the progressive stage improved mitochondrial structure and function in the hippocampus, reduced neurodegenerative alterations, prevented synaptic damage and neuronal loss, lowered Aβ burden and hyperphosphorylation of tau, and improved memory performance [118]. SkQ1 supports the therapeutic evidence that mitigating mitochondrial oxidative damage can slow downstream synaptic and neuropathological deterioration.

Despite encouraging preclinical findings, significant translational limitations remain. Many studies rely on transgenic or toxin-based models that do not fully recapitulate sporadic late-onset AD, and therapeutic efficacy may depend strongly on treatment timing (preclinical vs. symptomatic stages). Moreover, demonstrating antioxidant benefits in humans has historically been challenging, underscoring the need for improved biomarkers of mitochondrial oxidative stress, better patient stratification, and well-designed, robust trials aimed explicitly at testing disease-modifying outcomes.

### 3.2. Small Molecules

In addition to mitochondria-targeted antioxidants, several small molecules have been identified to modulate mitochondrial bioenergetics, dynamics, and signaling pathways implicated in AD. These compounds offer the advantage of favorable pharmacokinetics, blood–brain barrier penetration, and the ability to fine-tune mitochondrial function without the need for genetic manipulation.

#### 3.2.1. CP2

CP2, a small-molecule modulator of mitochondrial complex I, has emerged as a promising therapeutic candidate in AD models. Unlike classical mitochondrial inhibitors, CP2 exerts partial, controlled inhibition of complex I, enhancing mitochondrial efficiency by reducing excessive electron leakage and ROS production. Chronic CP2 treatment in 3xTg-AD mice restored synaptic activity, improved cognitive performance, normalized glucose metabolism, and enhanced energy homeostasis [119]. These benefits were accompanied by a significant reduction in hyperphosphorylated tau levels, mediated through increased activity of PP2A and suppression of tau-associated kinases such as Cyclin-dependent kinase 5 (CDK5) and GSK3β [119]. Consistent findings have also been reported in APP/PS1 mice, where CP2 treatment ameliorated AD-related pathology and improved cognitive outcomes, particularly in female mice. Importantly, mild complex I modulation by CP2 restored synaptic structure and normalized mitochondrial distribution within the hippocampus, showing its ability to improve both metabolic and synaptic defects without inducing explicit mitochondrial toxicity [120].

#### 3.2.2. Mdivi-1

Mdivi-1 is another class of small molecules that targets mitochondrial dynamics, particularly excessive fission mediated by dynamin-related protein 1 (DRP1). Mdivi-1, a DRP1 inhibitor, suppresses mitochondrial fission by interfering with DRP1 GTPase activity, thereby reducing mitochondrial fragmentation [78]. In neurodegenerative disease models, Mdivi-1 has been shown to prevent cytochrome c release, improve mitochondrial morphology, and attenuate disease-related phenotypes [42,121]. However, some studies suggest that Mdivi-1 may exert off-target effects, underscoring the need for improved specificity and careful interpretation of its neuroprotective actions [122].

#### 3.2.3. DDQ

Recent studies have identified diethyl (3,4-dihydroxyphenethylamino) (quinolin-4-yl) methylphosphonate (DDQ) as a novel mitochondrial modulator with multitarget activity. DDQ treatment improves the expression of mitochondrial and synaptic genes dysregulated in AD and enhances synaptic activity with reduced Aβ pathology [123,124]. DDQ also promotes a favorable shift in amyloid processing by reducing toxic Aβ _42_ levels while increasing Aβ _40_, a form linked with lower neurotoxicity [123].

#### 3.2.4. Resveratrol

In addition to these mitochondria-localized agents, broader antioxidant and redox-modulating compounds such as resveratrol have been studied in AD models and reported to reduce oxidative stress markers and improve memory performance in Aβ-challenged animals [125]. However, because such compounds are not specifically engineered for mitochondrial targeting, their inclusion is best framed as complementary evidence supporting oxidative stress modulation, rather than as primary examples of mitochondria-targeted antioxidants.

### 3.3. Gene Therapy Targets Mitochondrial Pathways

Gene therapy has emerged as a powerful strategy for modulating mitochondrial pathways implicated in neurodegenerative diseases, offering the potential for sustained and targeted correction of cellular dysfunction. While AD is not caused by single-gene mutations, mitochondrial gene therapy approaches aim to enhance mitochondrial resilience, bioenergetic capacity, and antioxidant defenses rather than directly targeting amyloid or tau pathology. Current strategies focus on nuclear-encoded mitochondrial regulators, as direct manipulation of mtDNA poses significant technical challenges [126]. Advances in mitochondrial gene editing, including mitochondrially targeted zinc-finger nucleases (mtZFNs), have demonstrated proof-of-concept efficacy in selectively eliminating mutant mtDNA and promoting repopulation with healthy copies in inherited mitochondrial disorders [127]. Although these approaches remain experimental, they establish a conceptual framework for correcting mtDNA instability and improving mitochondrial function in disease contexts.

#### 3.3.1. Adeno-Associated Virus (AAV)-Based Vectors

These vectors have become the preferred platform for mitochondrial gene delivery due to their favorable safety profile, long-term transgene expression, and neuronal targeting capabilities [128]. In AD models, AAV-mediated delivery of mitochondrial-protective genes has shown encouraging results. For instance, hippocampal delivery of AAV9-DJ-1 in APP/PS1 mice improved cognitive performance and activated key neuroprotective pathways, including NRF2 signaling, AMP-activated protein kinase (AMPK) phosphorylation, and autophagy-related mechanisms [129]. These effects were accompanied by enhanced antioxidant capacity and reduced oxidative stress, underscoring DJ-1’s role in maintaining mitochondrial redox homeostasis [129].

#### 3.3.2. PGC-1α

Another promising target is PGC-1α, a master regulator of mitochondrial biogenesis and oxidative metabolism. Lentiviral-mediated overexpression of PGC-1α in the hippocampus and cortex of APP23 mice resulted in improved spatial and recognition memory, reduced Aβ accumulation, and inhibition of β-secretase (BACE1) expression [130]. Additionally, PGC-1α gene delivery attenuated neuroinflammation, preserved pyramidal neurons in the CA3 region, and enhanced neurotrophic factor expression, showing its broad neuroprotective potential [130].

Despite these promising findings, several challenges limit the immediate clinical translation of mitochondrial gene therapy for AD. Efficient and widespread delivery across the blood–brain barrier, optimal timing of intervention, potential immune responses, and long-term safety remain key considerations. Moreover, given the heterogeneity of sporadic AD, gene therapy approaches are likely to be most effective when applied early in disease progression or combined with complementary therapies targeting metabolic and synaptic dysfunction.

### 3.4. Mitochondrial Permeability Transition Pore (mPTP) Inhibitors

Cyclophilin D (CypD): The mitochondrial permeability transition pore (mPTP) is a high-conductance channel whose pathological opening leads to mitochondrial membrane depolarization, collapse of ATP synthesis, and initiation of cell death pathways [131,132]. CypD, a mitochondrial matrix peptidyl-prolyl cis–trans isomerase, is a critical regulator of mPTP opening and sensitizes mitochondria to metabolic and oxidative stress. Increasing evidence implies dysregulated mPTP activity as a key contributor to mitochondrial impairment and neuronal vulnerability in AD. In AD, elevated CypD expression and increased formation of Aβ–CypD complexes have been detected in cortical and hippocampal mitochondria [133]. These interactions lower the threshold for mPTP opening, rendering mitochondria more susceptible to calcium overload and oxidative stress. Pathological mPTP opening results in loss of mitochondrial membrane potential, impaired oxidative phosphorylation, excessive ROS production, and release of pro-apoptotic factors, thereby linking mitochondrial stress directly to neuronal dysfunction and degeneration [134]. Genetic and pharmacological inhibition of CypD has demonstrated neuroprotective effects in AD models. CypD-deficient transgenic mice exhibit improved mitochondrial function, preserved ATP production, reduced oxidative damage, and enhanced learning and memory performance during aging and in AD-like conditions [133]. These findings support the concept that suppressing pathological mPTP opening can interrupt the cascade of mitochondrial failure and neuronal loss in AD. Pharmacological inhibition of CypD using cyclosporine A has been shown to prevent mitochondrial depolarization, limit cytochrome c release, and reduce tau burden and synaptic dysfunction in experimental models [135]. However, the clinical applicability of cyclosporine A is limited by its immunosuppressive effects and poor central nervous system specificity, highlighting the need to develop more selective mPTP inhibitors that target CypD without systemic toxicity. Beyond its role in mPTP regulation, CypD has been implicated in broader mitochondrial signaling and gene regulatory processes that influence mitochondrial bioenergetics and stress responses. Dysregulation of these functions may further contribute to mitochondrial instability and neurodegeneration in AD, although their precise relevance in human disease requires further investigation. These findings identify CypD-mediated mPTP opening as a critical node linking mitochondrial stress to irreversible neuronal injury in AD. Targeting pathological mPTP activation represents a promising strategy to preserve mitochondrial integrity and neuronal survival, particularly when combined with interventions that enhance mitochondrial bioenergetics and quality control. However, translating mPTP inhibitors into clinical practice will require improved drug specificity, optimized delivery, and careful patient stratification.

**Table 1:** The compounds targeting mitochondrial dysfunction in AD.

Compound	Effects in AD	References
mitoQ	Improved cognition, reduced oxidative stress and inflammation, and lowered Aβ levels.	[113,115,116,117]
SS-31	Stabilizes mitochondrial membrane, improves mitochondrial function, improves bioenergetics, and restores synaptic protein levels.	[136]
SkQ1	It improves mitochondrial function, memory, and prevents synaptic damage and neuronal loss.	[118]
Resveratrol	Reduced oxidative stress marker, improved memory.	[125]
CP2	Controlled complex I inhibition and reduced ROS generation, restored synaptic activity, and improved cognition.	[119,120]
mdivi-1	DRP1 inhibitor suppresses mitochondrial fission and improves mitochondrial morphology.	[42,78,121,122]
DDQ	Reduces DRP1–Aβ interaction, enhances synaptic activity, and reduces Aβ levels.	[123,124]
Gene therapy	Enhances antioxidant pathways (NRF2, AMPK), improves cognition. Improve mitochondrial biogenesis, reduce Aβ accumulation, and improve memory.	[126,127,128,129]
Cyclosporine A	Prevent mitochondrial depolarization, limit cytochrome c, and reduce tau cleavage and synaptic impairment.	[135]
Mitochondria transplant	Replaces damaged mitochondria, reduces Aβ levels, improves spatial learning and memory, and decreases neuronal loss and gliosis.	[137,138]
NAD+ precursors	Enhance mitophagy, improve mitochondrial resistance to oxidative stress, and reduce Aβ and tau burden.	[81,139,140]
Urolithin A	Induces mitophagy and improves mitochondrial function.	[141,142,143,144]
Spermidine	Promotes autophagy and mitophagy and reduces the oxidative burden.	[145,146,147,148,149]
Melatonin	Reduced oxidative stress increases the expression of antioxidant enzymes and stabilizes mitochondrial integrity.	[150,151,152]
NAC	Protect against ROS by restoring glutathione (GSH), reduced Aβ, and phosphorylated tau levels.	[153,154,155,156,157,158]
Photobiomodulation	Reduces Aβ and tau pathology, and improves cognition	[159,160]

### 3.5. Mitochondrial Transplantation

Mitochondrial transplantation, also known as mitotherapy, has emerged as a novel therapeutic strategy to restore mitochondrial function by directly providing healthy, functional mitochondria to cells with impaired bioenergetics. Unlike pharmacological or gene-based approaches that modulate specific pathways, mitotherapy offers a unique advantage by simultaneously targeting multiple aspects of mitochondrial dysfunction, including ATP production, calcium homeostasis, redox balance, and metabolic regulation [137]. In experimental models of AD, mitochondrial transplantation has shown promising neuroprotective effects [137]. Intravenous or intracerebral delivery of healthy mitochondria in AD rodent models improved spatial learning and memory, restored mitochondrial membrane potential, enhanced calcium buffering, and reduced oxidative stress [137]. These functional improvements were accompanied by decreased neuronal loss and gliosis in the hippocampus, as well as normalization of key mitochondrial enzyme activities, including citrate synthase and cytochrome c oxidase [138]. Notably, mitotherapy also reduces the accumulation of Aβ, suggesting that restoration of mitochondrial function may indirectly modulate classical AD pathology [138]. Importantly, mitotherapy addresses mitochondrial dysfunction at a systemic level rather than targeting individual molecular defects. By replenishing functional mitochondria, this approach bypasses impaired mitochondrial biogenesis, defective mitophagy, and dysregulated signaling pathways that limit the effectiveness of endogenous mitochondrial repair mechanisms in AD neurons. As such, mitotherapy may be particularly beneficial in advanced disease stages where intrinsic mitochondrial recovery processes are severely compromised. Despite its promise, several challenges currently limit the clinical translation of mitochondrial transplantation. These include optimizing mitochondrial isolation and preservation, ensuring efficient delivery across the blood–brain barrier, determining the longevity and functional integration of transplanted mitochondria, and addressing potential immunogenicity or off-target effects. Moreover, most evidence to date derives from small-animal models, and the feasibility, safety, and efficacy of mitotherapy in humans remain to be established.

### 3.6. Mitophagy-Enhancing Compounds

Given the accumulation of damaged mitochondria and impaired mitophagic flux in AD, pharmacological strategies that enhance mitochondrial quality control have gained increasing interest. Unlike mitochondria-targeted antioxidants that primarily mitigate oxidative damage, mitophagy-enhancing compounds aim to restore the selective clearance of dysfunctional or damaged mitochondria, thereby maintaining mitochondrial integrity, bioenergetic efficiency, and neuronal resilience.

#### 3.6.1. NAD^+^

One of the most extensively studied approaches involves restoration of intracellular nicotinamide adenine dinucleotide (NAD^+^) levels. NAD^+^ is a critical metabolic cofactor that regulates mitochondrial function, redox homeostasis, and autophagy through NAD^+^-dependent enzymes such as sirtuins and poly (ADP-ribose) polymerases. NAD^+^ levels decline with aging and are further reduced in AD models [81,139,140]. Supplementation with NAD^+^ precursors, including nicotinamide, nicotinamide mononucleotide (NMN), and nicotinamide riboside (NR), has been shown to enhance mitophagy, improve mitochondrial resistance to oxidative stress, decrease accumulation of Aβ and phosphorylated tau proteins, and prevent cognitive decline in AD mouse models. These benefits are primarily mediated through activation of Sirtuin 3 (SIRT3) and CREB-dependent transcriptional programs that support mitochondrial turnover and bioenergetics [140,161]. Beyond mitophagy, NAD^+^ restoration also supports mitochondrial biogenesis through SERT1-mediated activation of PGC-1α and contributes to DNA repair via PARP-dependent mechanisms, underscoring their broad mitochondrial-protective potential.

#### 3.6.2. Urolithin

It is a gut microbiota-derived metabolite of dietary ellagitannins, representing another promising mitophagy enhancer. Urolithin A has been shown to induce Mitophagy and improve mitochondrial function across multiple model systems [141]. In neurodegenerative disease contexts, urolithin A promotes mitochondrial turnover, improves ATP production, and reduces oxidative stress, thereby supporting neuronal survival [141,142]. Importantly, urolithin A has demonstrated promising safety and bioavailability profiles in humans, positioning it as a translationally attractive candidate for targeting mitochondrial dysfunction in aging-related disorders, including AD [143,144].

#### 3.6.3. Spermidine

It is a naturally occurring polyamine that promotes autophagy and mitophagy through epigenetic and metabolic mechanisms, including inhibition of histone acetyltransferases and activation of autophagy-related genes. Spermidine administration has been shown to restore mitochondrial homeostasis, protect against age-associated memory impairment, and improve neuronal survival in an autophagy-dependent manner [145,146,147]. By facilitating the clearance of damaged mitochondria, spermidine reduces the oxidative burden. It supports synaptic function, highlighting its potential as a dietary or pharmacological intervention to mitigate mitochondrial dysfunction in AD [145,148,149].

Thoroughly, NAD^+^ precursors, urolithin A, and spermidine converge on mitophagy and mitochondrial quality control pathways, offering a complementary therapeutic strategy to antioxidant and bioenergetic interventions. By promoting the selective removal of impaired or damaged mitochondria, these compounds address a fundamental pathogenic mechanism in AD. Their most significant therapeutic potential may lie in the early or preventive phase, where enhancing mitochondrial turnover might delay synaptic dysfunction and slow neurodegenerative progression.

### 3.7. SS-31

A second class of mitochondria-directed agents includes peptides that stabilize mitochondrial membranes and improve mitochondrial performance under stress. SS-31 (elamipretide) has been reported to have protective effects in APP/PS1 mice when administered around the onset of symptoms, delaying behavioral decline and reducing mitochondrial and synaptic dysfunction. Mechanistically, SS-31 improved mitochondrial function, reduced oxidative stress, improved fission–fusion imbalance, and partially reduced amyloid pathology while restoring synaptic protein levels [136]. These findings support the concept that targeting mitochondrial membrane stability and bioenergetics can support functional benefits in AD models.

### 3.8. Melatonin

Melatonin is an endogenous indoleamine primarily known for regulating circadian rhythms and for exerting neuroprotective effects through dual mitochondrial mechanisms: direct antioxidant activity and inhibition of the mPTP. Melatonin readily crosses the blood–brain barrier and accumulates within mitochondria, where it directly scavenges ROS and nitrogen species and enhances the expression of antioxidant enzymes. In AD models, melatonin treatment has been shown to preserve mitochondrial membrane potential, inhibit opening of the mitochondrial permeability transition pore, and suppress cytochrome c release, thereby protecting neurons from Aβ-induced apoptosis [150]. A study in transgenic AD mouse models demonstrates that chronic melatonin administration reduces oxidative stress, reduces neuroinflammation, lowers Aβ burden, and improves cognition [151]. Mechanistically, melatonin increases the expression of anti-apoptotic proteins, such as B-cell lymphoma 2 (Bcl-2), while suppressing pro-apoptotic factors, including Bcl-2-associated X protein (Bax), thereby stabilizing mitochondrial integrity and promoting neuronal survival [152]. These findings support melatonin’s role as a mitochondrial protectant rather than a direct disease-modifying agent.

### 3.9. N-Acetyl-Cysteine (NAC)

It acts primarily by replenishing intracellular glutathione (GSH), the most abundant endogenous antioxidant critical for mitochondrial redox homeostasis [158]. By restoring GSH levels, NAC enhances mitochondrial antioxidant capacity, inhibits oxidative damage to mitochondrial DNA and proteins, and improves mitochondrial function. Both in vitro and in vivo studies have demonstrated that NAC reduces Aβ and phosphorylated tau levels, attenuates oxidative stress, and improves learning and memory performance in AD animal models [153,154,155]. NAC has also been evaluated in clinical settings. Long-term administration of NAC-containing nutraceutical formulations in individuals with mild cognitive impairment or early AD has been associated with improvements in cognitive and behavioral outcomes [156,157]. However, definitive disease-modifying effects remain to be established [156,157]. These findings highlight NAC’s translational potential as an adjunct therapy to mitigate mitochondrial oxidative stress.

### 3.10. Photobiomodulation (PBM)

PBM is an emerging non-invasive therapeutic approach that uses red and near-infrared (NIR) light (600–1100 nm) to enhance mitochondrial function, with cytochrome c oxidase (Complex IV) as its primary biological target [162,163]. Since cytochrome c oxidase activity is reduced in AD brains, PBM directly addresses this bioenergetic deficit by promoting electron transfer, enhancing ATP production, and reducing oxidative stress [163,164]. In transgenic AD animal models, transcranial PBM has been shown to reduce Aβ plaque burden, attenuate tau hyperphosphorylation, restore mitochondrial membrane potential, and improve cognition [159,160,165]. Early pilot clinical studies in patients with mild cognitive impairment (MCI) and mild-to-moderate AD have reported improvements in cognition and cerebral blood flow, with a favorable safety profile [166]. Although variability in treatment parameters (wavelength, dose, and delivery method) and the limited number of large randomized controlled trials (RCTs) currently restrict clinical translation, PBM represents a mechanistically rational mitochondria-targeted strategy that complements existing therapeutic approaches for AD.

## 4. Conclusions

Mitochondrial dysfunction has emerged as an important contributor to the pathogenesis of AD, linking metabolic failure, oxidative stress, synaptic dysfunction, and neuronal loss. Although AD is a multifactorial disorder involving several interconnected pathological pathways, including amyloid and tau accumulation, neuroinflammation, and vascular dysfunction, growing evidence supports the mitochondrial cascade hypothesis, suggesting that mitochondria may act not only as downstream targets of neurodegeneration but also as active participants in disease onset and progression. Importantly, mitochondrial dysfunction in AD is multifaceted rather than pathway-specific, suggesting that effective therapeutic strategies must address mitochondrial health at a systemic level. Despite compelling mechanistic and preclinical evidence, translation of mitochondrial-targeted therapies into clinical benefit remains limited.

Future progress will depend on refining preclinical models, identifying robust biomarkers of mitochondrial dysfunction, and adopting precision-medicine approaches to stratify patients based on mitochondrial and metabolic phenotypes. Early intervention, combination therapies targeting complementary mitochondrial pathways, and integration with lifestyle and metabolic interventions may be essential to achieving disease-modifying effects. Advances in mitochondrial imaging, omics-based profiling, and targeted delivery technologies are likely to further accelerate this field. Thus, targeting mitochondrial dysfunction represents a promising and biologically grounded strategy for addressing the complex pathology of AD.

## Data Availability

No new data was analyzed during this study.
